# Improved charge carrier lifetime in planar perovskite solar cells by bromine doping

**DOI:** 10.1038/srep39333

**Published:** 2016-12-16

**Authors:** David Kiermasch, Philipp Rieder, Kristofer Tvingstedt, Andreas Baumann, Vladimir Dyakonov

**Affiliations:** 1 Experimental Physics VI, Julius Maximilian University of Würzburg, Würzburg, 97074, Germany; 2 Bavarian Center for Applied Energy Research e.V. (ZAE Bayern), Würzburg, 97074, Germany

## Abstract

The charge carrier lifetime is an important parameter in solar cells as it defines, together with the mobility, the diffusion length of the charge carriers, thus directly determining the optimal active layer thickness of a device. Herein, we report on charge carrier lifetime values in bromine doped planar methylammonium lead iodide (MAPbI_3_) solar cells determined by transient photovoltage. The corresponding charge carrier density has been derived from charge carrier extraction. We found increased lifetime values in solar cells incorporating bromine compared to pure MAPbI_3_ by a factor of ~2.75 at an illumination intensity corresponding to 1 sun. In the bromine containing solar cells we additionally observe an anomalously high value of extracted charge, which we deduce to originate from mobile ions.

Organometallic halide perovskite solar cells exhibit exceptionally good power conversion efficiency (PCE) values exceeding already 20% in the lab^1^. Being crystalline by nature, perovskite absorbers have the advantage of superior charge carrier transport properties leading to high charge carrier mobility as well as impressive diffusion lengths^2^,^3^,^4^,^5^. Nevertheless, its complex crystal structure and the composition of the crystal unit cell may result in lattice distortion and grain boundaries, which ultimately lead to scattering and trapping. The charge carrier lifetime is one of the most important key parameters in a solar cell. In combination with the charge carrier mobility, it defines the diffusion length and therefore the probability of a photogenerated charge carrier to reach the respective electrode. In general, defects in the crystal structure are expected to decrease the lifetime of photogenerated charge carriers in the absorber, as additional recombination pathways speed up the loss of excess carriers. Recent publications indicate that such defects are mostly located at the grain boundaries^6^,^7^ or at the surface of the perovskite crystal^8^, therefore morphology engineering by film processing and chemical composition can influence the charge carrier recombination in the absorber, helping to achieve high performance perovskite solar cells^9^,^10^,^11^. Most of the recombination studies presented so far have been focusing on perovskite films or crystals using photoluminescence (PL)^7^,^12^,^13^, Terahertz (THz) spectroscopy^14^ or microwave photoconductivity (TRMC)^14^,^15^,^16^, but only a few have studied charge carrier lifetimes incomplete solar cell devices^17^,^18^. It is important to note that not all experimental techniques probe the same lifetime related processes. In time-resolved PL measurements on films the radiative recombination of photogenerated charge carriers is often probed at high excitation densities, the TRMC is sensitive to fast charge carriers driven by high frequency electric fields and also laser pulse based technique, whereas in the transient photovoltage (TPV) method, the relevant bulk lifetime of photogenerated carriers defining the photovoltage of the solar cell is directly monitored.

Here, we report on charge carrier recombination kinetics in planar organometallic halide perovskite solar cells with varying degree of bromine content. Charge carrier kinetics are probed by combining TPV, identifying the charge carrier lifetime, and charge carrier extraction (CE), determining the charge carrier density in the device under identical and solar relevant conditions. The experimental techniques of TPV and CE have been implemented earlier to determine charge carrier recombination kinetics in thin film solar cells such as dye sensitized solar cells^19^, organic solar cells^20^,^21^,^22^,^23^,^24^ and just recently in mesoporous perovskite solar cells^18^. We herein focus our study on the impact of bromine content on charge carrier lifetimes and densities in planar halide perovskite solar cells. We reveal a direct correlation between the amount of bromine content and the charge carrier lifetime. The CE experiments provided an anomalously large signal at longer timescales in the solar cell with the most bromine content, leading to a recombination current J_*rec*_, which is larger than the short circuit current J_*sc*_ at 1 sun. We tentatively assign this to a possible contribution from mobile ions.

## Results and Discussion

**Figure 1:**
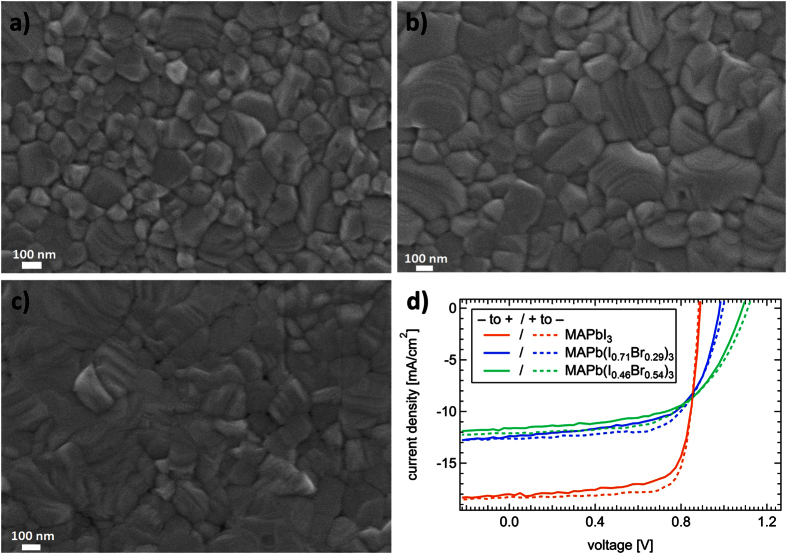
SEM images (**a–c**) and photovoltaic performance (**d**) of different perovskite layers. (**a**) Shows MAPbI_3_, (**b**) represents MAPb(I_0.71_Br_0.29_)_3_ made via a MABr precursor. In (**c**) the bromine content was increased using PbBr_2_ instead of MABr.

To study the impact of different bromine-to-iodine ratios, we varied the precursors in the well-established interdiffusion approach. Starting from PbI_2_ and methylammonium iodine (MAI), bromine was introduced in the perovskite lattice by replacing the salts with MABr or PbBr_2_ (MAPb(I_1−*x*_Br_*x*_)_3_). More details on the sample configuration and preparation can be found in the experimental section. The effect of different precursor solutions on film topography and surface crystallinity was first analyzed by means of scanning electron microscope (SEM). Figure 1a–c) shows the surfaces of MAPbI_3_ (a), MAPb(I_1−*x*_Br_*x*_)_3_ (MABr) (b) and MAPb(I_1−*x*_Br_*x*_)_3_ (PbBr_2_) (c). The images reveal smooth perovskite layers consisting of individual crystals with diameters between sub *μ*m and 500 *μ*m, rendering an almost pinhole-free film. From x-ray diffraction (XRD) measurements (Supplementary Information, Fig. S1) we clarified the impact of different precursor solutions on the bromine content. In comparison to pure MAPbI_3_ the lattice distance is decreased by using MABr and PbBr_2_. Thereby, the approach with PbBr_2_-precursor contains the highest bromine-to-iodine ratio^25^. According to Noh *et al*. and Gil-Escrig *et al*. the bromine content in the mixed halide perovskites was identified by determining the band gap of the layers via optical absorption measurements (Supplementary Information, Fig. S2)^26^,^27^. In case of MAPb(I_1−*x*_Br_*x*_)_3_ (MABr) x was calculated to be 0.29, whereas for MAPb(I_1−*x*_Br_*x*_)_3_ (PbBr_2_) x was determined to be 0.54. Therefore these layers will be abbreviated as MAPb(I_0.71_Br_0.29_)_3_ and MAPb(I_0.46_Br_0.54_)_3_. The photovoltaic performance of every composition was analyzed by measuring the J–V characteristics. The perovskite layer was sandwiched between planar transport materials, namely PEDOT:PSS acting as a hole selective layer and a combination of PC_60_BM, C_60_ and BCP operating as electron selective layer sequence and Au as top electrode. Fig. 1d) displays the J–V curves for the best performing devices measured with a scan speed of 463 mV/s under AM1.5 G illumination. All solar cells display a very small hysteresis, hence, the PCE of forward and backward scans are comparable and show a decent performance with a PCE between 8–13% for all studied devices.The impact of bromine doping is evident in an increased V_*oc*_ from 0.882 V to 1.113 V which can mainly be assigned to the increased band gap of the bromine containing cells^28^. As J_*sc*_ decreased simultaneously from 18.3 mA/cm^2^ (MAPbI_3_) to 12.1 mA/cm^2^ (MAPb(I_0.46_Br_0.54_)_3_) (parameters from the reverse scan) the overall PCE shows a slight decrease. Note that further optimization of the corresponding perovskite layer thickness may lead to an increase in J_*sc*_. Here, we deliberately used the same parameters for the cell preparation.

**Figure 2:**
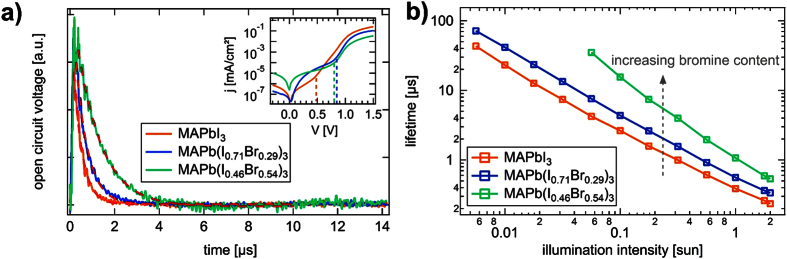
(**a**) TPV decays at a background illumination intensity of 1 sun for the three different devices measured at 300 K. The single exponential fits are shown as dashed lines. Inset: dark current of the corresponding solar cells shown in a log-lin. plot. The transitions from the shunt-limited region to the diode-like exponential behavior are marked with dashed lines. (**b**) Calculated TPV lifetimes as function of the illumination intensity.

To study the charge carrier recombination dynamics, the techniques of TPV and CE are applied according to the methodology previously outlined^22^. Normalized TPV transients for the three devices are plotted in Fig. 2a), measured under illumination conditions equivalent to 1 sun. To determine the small perturbation decay lifetime, the signal was fitted with a single exponential function. However, there is now an ongoing discussion whether a single^5^,^10^,^29^ or double^18^,^30^,^31^,^32^,^33^,^34^ exponential fit should be used in perovskite solar cells. We note, that almost all groups using the double exponential function have been investigating mesoporous device layouts, which had titanium dioxide (TiO_2_) in common. This could be an indication that the origin of a decay with two characteristic lifetimes should be assigned to presence of a TiO_2_ scaffold and not necessarily to the perovskite itself. Indeed, Lee and coworkers attributed the fast component to recombination in the active layer and the slow component to a recombination pathway in TiO_2_^33^. It is also conceivable that the charge transport in the TiO_2_ layer is responsible for the slow characteristic time^32^, since it is demonstrated that transport occurs faster in perovskite compared to TiO_2_^35^. In our measurements on planar perovskite solar cells without TiO_2_ scaffold, we did not observe two time components over two orders of magnitude in voltage and the data could always be fitted with a single exponential function accordingly (red dashed lines in Fig. 2a)). In case of MAPbI_3_, the lifetime under 1 sun illumination background intensity can be calculated to 0.39 *μ*s, for MAPb(I_0.71_Br_0.29_)_3_ it is 0.56 *μ*s and for MAPb(I_0.46_Br_0.54_)_3_ it is 1.07 *μ*s. The inset of Fig. 2a) shows the dark current for the three devices in a semi-logarithmic plot. Clearly, the MAPbI_3_ device obeys the highest shunt resistance of all investigated solar cells with transition from the shunt to the exponential regime around 500 mV. It is important to note that TPV experiments should be conducted in the exponential diode regime of a solar cell to correctly study the bulk material properties of the active layer, instead of recombination losses via parasitic shunt pathways^36^. Here, we conducted measurements in the range of 0.0056 up to 2 suns accordingly by changing the current of the LED and using different neutral density filters. This light intensity span resulted in corresponding V_*oc*_ values ranging from 692 mV to 856 mV for the MAPbI_3_ device, clearly being in the exponential diode regime. In contrast, for MAPb(I_0.46_Br_0.54_)_3_ we also obtained open circuit voltages already affected by the shunt regime (<800 mV) in the lower illumination intensity range and hence excluded them from evaluation. Figure 2b) displays the calculated charge carrier lifetime values *τ* in dependence on the applied illumination intensity. As expected, the increase in charge carrier density n leads to a decrease in *τ*. This is valid for all three investigated types of solar cells. For MAPbI_3_, *τ* is found to be in the range of 0.2–43.2 *μ*s. Studying MAPbI_3_ solar cells, Xiao *et al*. reported the lifetime of 1.7 *μ*s under 0.3 sun illumination^29^, which is very close to values obtained in this work for MAPbI_3_ under the same conditions. By bromine doping of the perovskite absorber, the charge carrier lifetime increases in the studied illumination intensity range: 0.36–72.5 *μ*s for MAPb(I_0.71_Br_0.29_)_3_ and 0.53–35.02 *μ*s for the MAPb(I_0.46_Br_0.54_)_3_ device.

To assess the possible impact of domain size on *τ*, additional measurements were performed using ammonium chloride (NH_4_Cl) and 1,8-diiodooctane (DIO) as additives in the PbI_2_-solution^37^,^38^ to slow down the chemical reaction with MAI and increase the domain size. In this case only the domain size of MAPbI_3_ is increased without changing the lattice structure. A top view SEM image of the resulting layer is provided in the Supplementary Information (Fig. S3), showing the enhanced size of the crystal domains. The results of TPV measurements on these devices are summarized in Fig. S4 in the Supplementary Information, showing an increase in *τ* with crystal domain size with a factor of ~1.18 at 1 sun. As depicted in Fig. 1a–c), the bromine-to-iodine ratio leads to a small increase in crystal domain size. By adding bromine to the perovskite lattice using PbBr_2_, the lifetime at 1 sun is increased by a factor of ~2.75, which is significantly higher than the impact solely of crystal domain size. Two possible explanations for the increased charge carrier lifetime may come into mind. Due to the bromine doping of the MAPbI_3_ the number of deep trap states which act as recombination centers decreases with increasing Bromine content. In this case the effective charge carrier lifetime which is the sum of all present recombination mechanisms is increased due to a decrease in Shockley-Read-Hall (SRH) recombination. Second, which is more likely the case, due to an incorporation of Bromine into the MAPbI_3_ lattice more defects are created which are shallow in nature acting as dopants for the MAPbI_3_ lattice. In contrast to deep trap states in the middle of the band gap, shallow traps close to conduction or valence band of the semiconductor partly trap charge carriers which has to be then released back into the band before they can recombine. In this case, the effective charge carrier lifetime would be increased as the event of trapping-and-release will slow down the actual recombination process leading to a reduced second order recombination. However, we note that from the given set of experiments we cannot identify the real origin of the increased charge carrier lifetime. Very recently, Yang *et al*. have demonstrated that by MABr treatment of a MAPbI_3_ film with bad quality leads to an improvement of the crystallinity and the overall PCE^39^. The MABr-treated solar cells obeyed improved charge collection and surface passivation properties possibly related to reduced defect states. This strongly supports our experimental observations.

**Figure 3:**
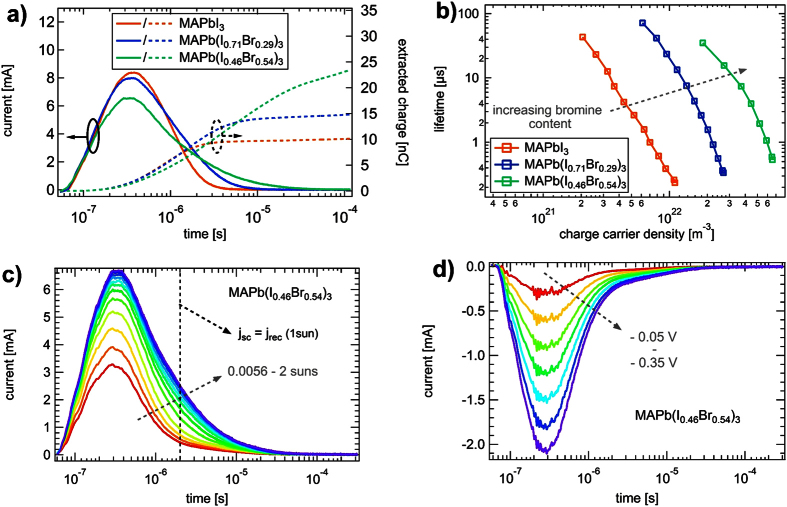
(**a**) CE signal (left axis) and the integral (right axis) for the three investigated devices after illuminated with 1 sun. (**b**) TPV lifetime plotted versus the charge carrier density determined with CE. (**c**) CE signal of MAPb(I_0.46_Br_0.54_)_3_ for different illumination intensities. (**d**) CE signal in the dark realized by applying negative voltages before switching to short circuit conditions.

Performing CE experiments at the same conditions as TPV allows us to determine the corresponding charge carrier density *n* at each background illumination intensity, and subsequently correlate it with the charge carrier lifetimes *τ*. This is possible, as the measurements were conducted at the same illumination intensities and the charge carrier density is directly correlated to V_*oc*_^17^. Figure 3a) summarizes the CE current signals (solid lines) and their respective integrals (dashed lines) for the studied solar cells. The devices were illuminated with 1 sun for a certain time (< 1 s) to reach steady state conditions before extracting the photogenerated charge carriers. The obtained charge carrier lifetimes from TPV plotted versus the carrier density from CE is shown for the three perovskites in Fig. 3b). The lifetime decreases with increasing n as expected for second order recombination and often reported in literature at high illumination intensities^14^,^30^. Under 1 sun illumination, the charge carrier density was calculated to be 9.4 ⋅ 10^21^ m^−3^ in case of MAPbI_3_, 2.4 ⋅ 10^22^ m^−3^ for MAPb(I_0.71_Br_0.29_)_3_ and 5.9 ⋅ 10^22^ m^−3^ for MAPb(I_0.46_Br_0.54_)_3_. Recently, O’Regan *et al*. reported on unusual charge carrier density obtained using the CE technique in mesoporous MAPbI_3_ solar cells^18^. The authors identified a large extraction current at time scales of several seconds, which would correspond to a very high amount of charges stored in the device, attributing it to a possible contribution by mobile ions. Here, we did not observe such a behavior, neither for our planar MAPbI_3_ or MAPb(I_0.71_Br_0.29_)_3_ device configuration. The charge extraction for these devices is completed already after 10^−5^ s to 10^−4^ s and further integration does not lead to any increase in the amount of extracted charges. However, MAPb(I_0.46_Br_0.54_)_3_ does display a much longer signal and the corresponding slope of the integral value gets approximately constant over several orders of magnitude in time (see Fig. 3a)). Moreover, with increasing bromine content, the charge extraction velocity is decreased, as the peak height is reduced and the extraction is slowed down. Following O’Regan *et al*., we subsequently calculated the recombination current J_*rec*_, taking into account the extracted charge Q(V_*oc*_), the corresponding lifetime τ(V_*oc*_) from TPV and the slope s from the τ(V_*oc*_) versus n(V_*oc*_) plot (Fig. 3b))^18^. At V_*oc*_ the charge generation rate equals the recombination rate. As no external current is flowing out of the device at V_*oc*_, the recombination current J_*rec*_ must be equal to the photocurrent J_*ph*_, which is best approximated by the short circuit current J_*sc*_. For the MAPbI_3_ device, all data points can be fitted by a constant slope s of 3.1. In contrast, for the mixed halide perovskites, s itself is strongly charge carrier density dependent. Thus, for both bromine containing perovskites we instead focus exclusively on the higher illumination intensities to be able to make conclusions at least at around real operating conditions. Here, slopes over 5 for MAPb(I_0.71_Br_0.29_)_3_ and MAPb(I_0.46_Br_0.54_)_3_ are obtained. Calculating J_*rec*_ from the present data finally leads us to following conclusions: for MAPbI_3_ J_*rec*_ is comparable to J_*sc*_ as expected under open circuit conditions^18^. In case of MAPb(I_0.71_Br_0.29_)_3_ and of MAPb(I_0.46_Br_0.54_)_3_ the determined J_*rec*_ is 50% higher than J_*sc*_ for 1 sun, which seems to be rather unphysical. Under 1 sun illumination intensity 33 nC are extracted after 1 ms for MAPb(I_0.46_Br_0.54_)_3_ (not corrected by the capacitance of the cell), which corresponds to the capacitance of cell) which corresponds to an anomalously large charge carrier density of around 2 ⋅ 10^23^ m^−3^. This is already around 7 times as large as that of the MAPbI_3_ device. To further analyze this unexpected behavior we take a closer look at the actual CE signal. Fig. 3c) summarizes the CE signals for MAPb(I_0.46_Br_0.54_)_3_ for several illumination intensities from 0.0056–2 suns. We note that the extraction peak is located at the same position for all intensities. After a first fast initial decay (3 ⋅ 10^−7^ s–2 ⋅ 10^−6^ s) however, a second slower extraction starts to become apparent. We calculated the time needed for the charge extraction to be finished in order to satisfy the requirement J_*sc*_ ≈ J_*rec*_. This time is marked in Fig. 3c) by the black dashed line. Interestingly, it hits exactly the time range where the slow component starts to be visible. Furthermore, taking Fig. 3a) into account, it becomes obvious that it also fits to the time range when the extraction is completed for the MAPbI_3_ device. The long timescale of the second extraction part therefore indicates a contribution distinct from electrons and holes. To examine its origin, we performed CE measurements in the dark by applying a reverse bias in the range from −0.35 V up to −0.05 V and extracted the charge by switching to J_*sc*_. At these voltages a negligible current is flowing through the device. The obtained CE signal shown in Fig. 3d) results from the charges stored on the electrode due to applied reverse bias. After a first initial peak, a slow component again starts to be dominant in the signal at ~2 *μ*s. The fact that this occurs at the same time as in the CE signals under illumination indicates that it has the same origin. We also revealed that the tail of the CE signal is visible in the complete range of illumination intensities and the dark and is therefore not caused by the photogenerated charge carriers themselves. From these studies, we conclude that photogenerated electrons and holes are responsible for the CE signal before 2 *μ*s, whereas another process causes the tail of the CE signal at longer time scales. Here, we want to exclude the effect of dipole reorientation leading to this tail. Chen *et al*. demonstrated that the characteristic relaxation times for the rotation of the organic cation are in the order of picoseconds, which is contrary to the observed timescales in CE (>10^−7^ s)^40^. However, a more likely explanation for this behavior can be the migration of mobile ions. It has been shown by Eames *et al*. that iodide ion vacancies may lead to ionic transport in the perovskite resulting in phenomena like the often observed current-voltage hysteresis^41^. Including it to the CE signal, which originated from photogenerated electron and holes only may lead to an overestimation of the extracted charge carrier density as observed by O’Regan and coworkers^18^. We point out that we also do not exclude a slow time component in the MAPbI_3_ device, but its impact is much less compared to the bromine devices.

## Conclusion

To conclude, we performed TPV and CE measurements on planar organometallic halide perovskite solar cells with varying bromine doping by using different precursor materials during the interdiffusion approach. We observed an increased charge carrier lifetime from 0.39 *μ*s for the MAPbI_3_ device to 1.07 *μ*s for MAPb(I_0.46_Br_0.54_)_3_ at an illumination intensity of 1 sun. We give two possible explanations for the lifetime increase. First, the decrease of number of deep trap states leading to less SRH recombination centers. Second, which is more likely, bromine act as dopants introducing shallow defects to the MAPbI_3_ lattice which may lead to a reduced second order recombination and hence increased carrier lifetime due to carrier trapping-and-release events. From CE measurements we observed a discrepancy between the recombination current J_*rec*_ and J_*sc*_ of the solar cell with the largest bromine content. Whereas the pure MAPbI_3_ solar cell displays a CE signal which is saturated after 0.1 ms, leading to a J_*rec*_ being comparable to J_*sc*_, the device with largest bromine content shows an anomalous behavior. The extracted charge is still increasing after 1 ms leading to an anomalously large value for the extracted charge carrier density, which disagrees with the expected value according to J_*sc*_. We revealed this continuously growing CE signal being also visible in the dark at reverse bias. In this case the solar device is acting as a capacitor and only charge carriers stored on the electrodes are detected. We assign this to ion migration due to the externally applied electric field contributing to the extracted charge signal, which cannot be observed in the pure MAPbI_3_ device.

## Methods

Solar cells are fabricated on indium tin oxide (ITO) covered glass substrates. Poly(3,4-ethylenedioxythiophene): poly(styrenesulfonate) (PEDOT:PSS) was spin coated to form a hole transport layer (HTL) with a thickness of around 35 nm. Perovskite films were synthesized with the well-known two-step interdiffusion process^42^. In case of MAPbI_3_, we used PbI_2_ (400 mg/ml) dissolved in N,N-Dimethylformamide (DMF) and CH_3_NH_3_I (MAI) (40 mg/ml) dissolved in 2-Propanol (2-Prop.). After spin-coating the lead-salt solution, the substrate was annealed for 15 minutes at 70 °C. Afterwards, MAI was spin-coated and heated at 100 °C for 90 minutes to form the active layer. To introduce bromine into the perovskite crystal, MABr and PbBr_2_ were used. Kulkarni *et al*. already have demonstrated the possibility of band-gap tuning with different precursor solutions^43^. In order to vary the bromine-to-iodine ratio in the film, two different batches of solar cells were synthesized: First, a 1:1 mixture (molar ratio) of MAI/MABr (20 mg/ml in 2-Prop.) was applied instead of pure MAI on top of the dried PbI_2_ layer. Such devices are referenced as MAPb(I_0.71_Br_0.29_)_3_ (the bromine-to-iodine ratio was determined by optical absorption measurements, Supplementary Information Fig. S2). For the purpose of increasing the bromine content, we used PbBr_2_ (400 mg/ml in Dymethylsulfoxide, DMSO) instead of PbI_2_. Then, the second step was carried out analogously as for MAPbI_3_. Solar cells made with this approach are abbreviated as MAPb(I_0.46_Br_0.54_)_3_. After forming the perovskite layer, PC_60_BM was spin casted from 1,2-Dichlorbenzene (DCB) solution (20 mg/ml) followed by 60 minutes annealing at 100 °C. In the last step, the substrates were transferred into an evaporation chamber in order to apply C_60_, Bathocuproin (BCP) and gold (Au) layers.

Current–voltage characterization was performed under inert atmosphere using a Keithley 237 source measure unit (SMU) and a AM1.5 G solar simulator (LOT-Oriel) which is calibrated to 100 mW/cm^2^ (1 sun).

Transient photovoltage (TPV) measurements were realized in a closed helium contact gas cryostat without exposure to ambient air using a 10 W white light LED for bias light illumination. The solar cells were kept under open circuit conditions using a 1.5 GΩ impedance. A pulsed Nd:YAG laser (*λ* = 532 nm excitation pulse, 80 ps) is providing a small optical perturbation generating additional charge carriers in the device. The voltage transient was then recorded by a digital storage oscilloscope (Agilent Infinium DSO90254A).

Charge carrier extraction (CE) measurements were performed in the same cryostat directly after TPV. The premeasured open circuit voltage is applied to the solar cell using a double pulse generator (Agilent 81150 A) under constant LED illumination. Triggered by the double pulse generator, the LED was switched off by shortening the constant current source (Keithley 2602) with a high power transistor. The resulting current transient was monitored via the digital storage oscilloscope mentioned above. The integrated charge was then corrected by the capacitance effect. To calculate the charge carrier density, a Veeco Dektak 150 Profilometer has been used for determining the active layer thickness. In order to vary the background illumination intensity over three order of magnitudes (0.0056 suns −2 suns) the LED current was controlled in combination with different neutral density filters. 1 sun equivalent is defined by the LED current matching the same short circuit current density Jsc of a solar cell as measured previously with the sun simulator.

## Additional Information

**How to cite this article**: Kiermasch, D. *et al*. Improved charge carrier lifetime in planar perovskite solar cells by bromine doping. *Sci. Rep.* **6**, 39333; doi: 10.1038/srep39333 (2016).

**Publisher’s note:** Springer Nature remains neutral with regard to jurisdictional claims in published maps and institutional affiliations.

## Supplementary Material

Supplementary Information
