# Supplementary material for: Improved charge carrier lifetime in planar perovskite solar cells by bromine doping
Source: Sci Rep. 2016 Dec 16;6:39333. doi: 10.1038/srep39333 (PMC5159823; doi:10.1038/srep39333)
Supplement: Supplementary Information [file srep39333-s1.pdf]

Supporting Information

Improved charge carrier lifetime in planar perovskite solar cells by bromine doping

David Kiermasch, Philipp Rieder, Kristofer Tvingstedt, Andreas Baumann, Vladimir Dyakonov

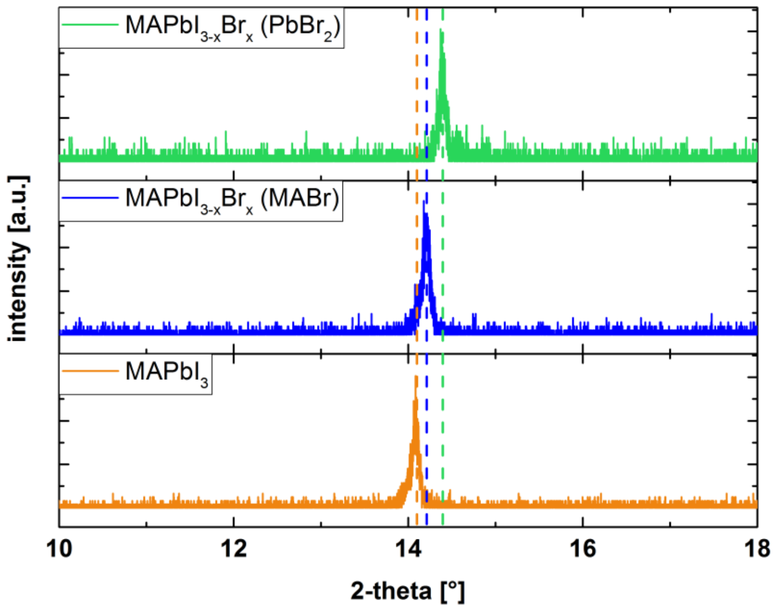

Figure S1. XRD spectra of mixed halide perovskite layers using PbI_2 + MAI (orange), PbI_2 + MAI/MABr (blue) and $PbBr_2$ + MAI (green) as precursor salts. Introducing bromine in the perovskite crystal leads to a decreased lattice constant.

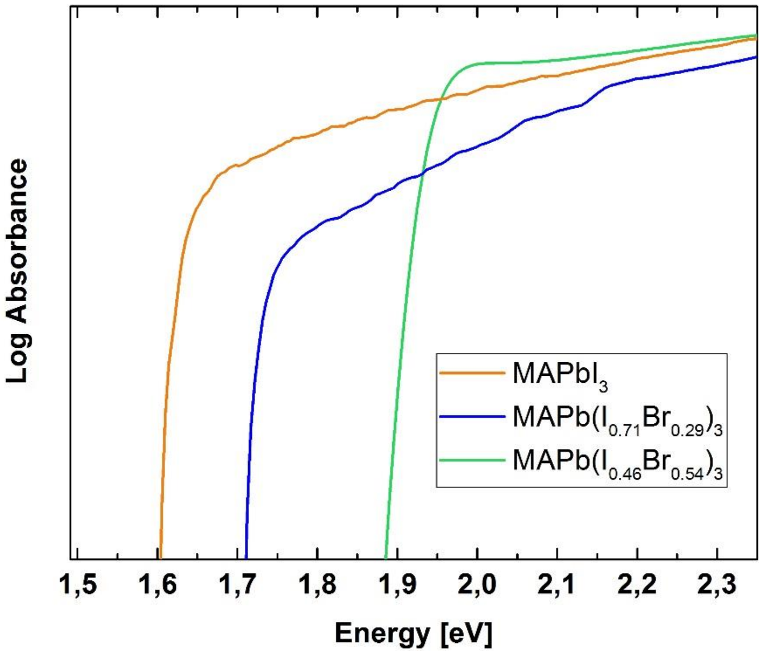

Figure S2. Optical absorption spectra for all studied perovskite films. According to Noh et al. and Gil-Escrig et al. we determined the band gap and estimated the bromine-to-iodine ratio for the $MAPb(I_{1-x}Br_x)_3$ layers[1,2]. For the PbI_2 + MAI/MABr approach we calculated x to be 0.29. In case of $PbBr_2$ + MAI the bromine content was increased up to 0.54.

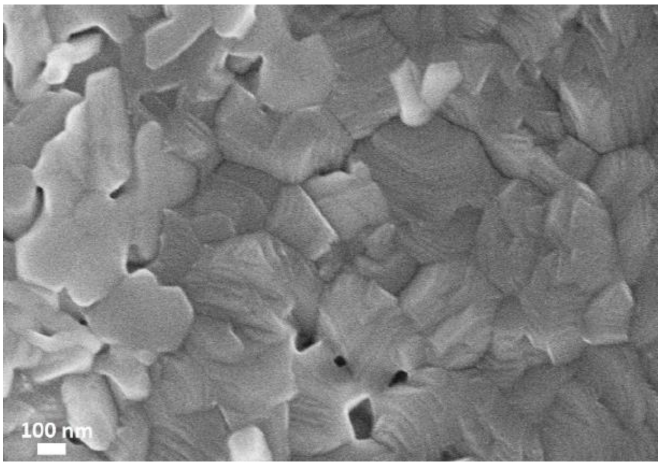

Figure S3. Surface SEM image of the resulting perovskite layer using NH_4Cl and DIO as additives in the PbI_2-solution. The additives lead to an increased crystal domain size without changing the lattice structure by slowing down the chemical reaction.

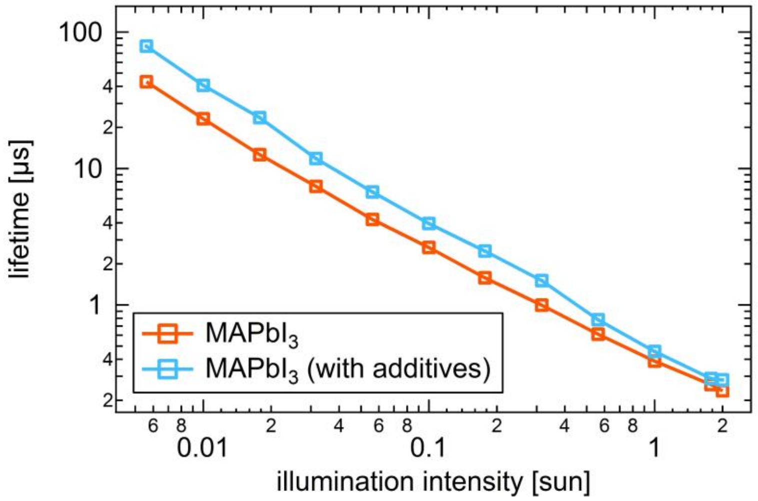

Figure S4. Impact of crystal domain size (varied by adding NH_4Cl and DIO to the PbI_2-solution) on charge carrier lifetimes measured with TPV. An increase in τ with crystal domain size by a factor of 1.18 at 1 sun illumination can be observed.

References

[1] Noh, J. H., Im, S. H., Heo, J. H., Mandal, T. N. & Seok, S. I. Chemical Management for Colorful, Efficient, and Stable Inorganic−Organic Hybrid Nanostructured Solar Cells. Nano Lett. **13**, 1764-1769 (2013)

[2] Gil-Escrig, L., Miquel-Sempere, A., Sessolo, M. & Bolink, H.J. Mixed Iodide-Bromide Methylammonium Lead Perovskite-based Diodes for Light Emission and Photovoltaics. J. Phys. Chem. Lett. **6**, 3743-3748 (2015)
